# The Impact of Histologic Variants on FSGS Outcomes

**DOI:** 10.1155/2014/913690

**Published:** 2014-10-29

**Authors:** Kristin Meliambro, Monica Schwartzman, Paolo Cravedi, Kirk N. Campbell

**Affiliations:** Division of Nephrology, Icahn School of Medicine at Mount Sinai, New York, NY 10029, USA

## Abstract

Focal segmental glomerulosclerosis (FSGS) is the most common glomerular disease leading to end-stage renal disease. The clinical course is highly variable with disparate responses to therapeutic intervention and rates of progression. Histologic variant subtype has been commonly used as a prognostic and therapeutic guide in the clinical management of FSGS. The tip lesion is widely considered to portend the most favorable prognosis and to be the most responsive to steroid therapy. Conversely, the collapsing lesion, more prevalent in patients of African descent, is associated with steroid resistance and higher risk of disease progression. In the 10 years since the Columbia classification system for FSGS was published, some retrospective and one prospective study explored the impact of histologic variants at the time of biopsy on FSGS outcomes. The results largely validate its clinical predictive value with respect to treatment response, though its utility in cases recurring after kidney transplantation is still unknown. Sampling and interpretation errors are additional sources of caution. More research is needed to fully define reproducible prognostic and therapeutic markers for this polymorphic disorder.

## 1. Introduction

Focal segmental glomerulosclerosis (FSGS) is increasing in incidence globally as a primary cause of end-stage renal disease (ESRD) [1, 2]. The spectrum of FSGS encompasses disease entities characterized by podocyte injury with foot process effacement leading to the characteristic histological pattern of obliteration of glomerular capillaries by extracellular matrix (ECM) accumulation [3]. Two frameworks for the classification of FSGS can be described. The first is etiologic which is distinguished amongst genetic, adaptive (postadaptive), virus-associated, drug-induced, and primary (idiopathic) types [4]. Genetic FSGS has been associated with mutations in over 20 genes encoding a range of molecules which appear to be critical for podocyte function [5–11]. Adaptive FSGS arises due to a mismatch between glomerular blood flow and glomerular filtration surface, leading to podocyte stress, detachment, and loss. Virus-associated FSGS includes viruses such as HIV and EBV and may occur via direct viral infection of the podocyte, circulating viral proteins, or as a consequence of the inflammatory cytokines released by other infected cells that interact with podocyte receptors [12]. Drug-induced FSGS is associated with a short list of medications including those that act on the podocyte (pamidronate, interferon-alpha) and those that damage the tubulointerstitium (e.g., lithium, cyclosporine, and tenofovir) [4]. Primary FSGS patients represent the majority of patients with the disease and are thought to display immune and/or cytokine abnormalities that lead to podocyte injury. This provides the rationale for the use of glucocorticoids as initial treatment.

A second histologic framework is provided by the Columbia classification scheme published in 2004 that describes five distinct FSGS variantsbased on light microscopic patterns [13]. This classification system can be applied to both primary and secondary forms of FSGS and has been widely used over the past 10 years both as a diagnostic and as a prognostic clinical tool. We will review herein the evidence of the prognostic value of the Columbia FSGS histologic classification in primary and recurrent forms after transplantation.

## 2. FSGS Classification Scheme

In 2000, an international panel of renal pathologists with mutual interest in FSGS convened at Columbia University, New York, NY, to achieve uniformity in pathologic interpretation of the histological variants of FSGS. The Columbia Working Classification of FSGS was published in 2004 [13] and divides these into perihilar, cellular, collapsing, tip lesion, and not otherwise specified (NOS) morphologic forms (Table 1).

The perihilar variant is defined by the presence of at least 1 glomerulus with perihilar hyalinosis with or without sclerosis with >50% of affected sclerotic glomeruli possessing these perihilar lesions [13].* Tip lesion, collapsing, and cellular variants must be excluded* [13]. This form has been described in both primary FSGS and secondary adaptive forms stemming from nephron loss or glomerular hypertension (i.e., due to obesity, reflux nephropathy, hypertension, sickle cell disease, etc.), usually accompanied by glomerular hypertrophy. The cellular variant is defined by identification of at least 1 glomerulus with endocapillary hypercellularity (including foam cells, macrophages and other leukocytes, and endothelial cells, occasionally associated with hyalinosis, karyorrhexis, and fibrin) involving >25% of the glomerular tuft, leading to occlusion of the capillary lumen [13].

This is the least common variant [15] and though foam cells may be seen in other FSGS subtypes, the diagnosis of cellular FSGS requires exclusion of tip lesion and collapsing variants [13]. Diagnosis of tip variant FSGS requires at least 1 segmental lesion involving the “tip” domain (the outer portion of the glomerular tuft next to the origin of the proximal tubule) with either ECM adhesion or confluence of podocytes with parietal or tubular epithelial cells at the tubular lumen or neck [13]. Most cases of tip lesion FSGS are primary/idiopathic in etiology and predominate in white adults [21]. Collapsing FSGS must be excluded, and the presence of perihilar sclerosis and/or endocapillary hypercellularity in any glomerulus also rules out tip variant [13]. Collapsing FSGS is characterized by the presence of at least 1 glomerulus with collapse and overlying podocyte hypertrophy and hyperplasia [13]. Most cases are either idiopathic in origin or HIV-associated and are more commonly found in black patients [22, 23]. Finally, FSGS NOS applies to a renal biopsy that does not meet the criteria for any other variant with findings of focal and segmental consolidation of the glomerular tuft by increased ECM, leading to obliteration of glomerular capillary lumen [13]. This is the most common subtype, and interestingly it has been observed from repeat biopsies that other variants may evolve into FSGS NOS over time [3, 21].

A recent study examined the ability of renal pathologists to classify FSGS according to the Columbia Working Classification [24]. Sixty-one digital images of individual glomeruli with FSGS were classified independently by six specialist renal pathologists. Agreement for 366 diagnoses by six observers was 75% with a kappa value of 0.676 (where *κ* = 1 means complete agreement; *κ* = 0 means random coincidental agreement). Six out of six observers agreed in 31 out of 61 cases (51%) and four or more in 53 cases (87%). These data suggest good interobserver reproducibility of the classification system, provided that biopsies are read by well-trained pathologists. However, difficulties may arise in the interpretation of lesions with mixed features of more than one Columbia type of FSGS in the same tissue specimen where the subjectivity of distinction between cellular and NOS further complicates the differential diagnosis.

## 3. Clinical, Therapeutic, and Prognostic Implications of Histological Variants

Since the introduction of the Columbia Working Classification, several retrospective studies and one prospective study have examined its prognostic and therapeutic implications. Their conclusions can be summarized in the context of correlations with demographics, overall disease severity, and clinical outcomes (Table 2).

### 3.1. Patient Demographics

The FSGSCT (FSGS clinical trial), a recent prospective study of 138 young ethnically diverse patients with steroid-resistant primary FSGS, found FSGS NOS to be the most common variant identified (68%), followed by collapsing FSGS (12%) and tip lesion FSGS (10%), with significantly less representation of perihilar (7%) and cellular (3%) variants [20, 25]. Within the NOS subgroup, most patients were children aged 2–12 years, while the collapsing subgroup tended to be older at disease onset (median age 16.5 years), and tip variant patients presented at an intermediate age (median age 15 years) between that of NOS and collapsing subgroups [20].

While the FSGS NOS subtype is the most common overall, the tip variant FSGS has been noted predominantly in Caucasian adults and collapsing FSGS in individuals of African descent [13]. A study by Deegens et al. that included a largely native Dutch population lacking black patients found tip lesion to be the most common FSGS variant (37% of patients) while collapsing lesions were rare (5%) [19].

In contrast, in a 41 patient cohort (1–18 year olds) in New Orleans, where 80.5% were black, no patients had the tip lesion [18]. It is notable in this study that while FSGS NOS was the most common variant (44% of cases), collapsing and cellular lesions accounted for 24% and 32%, respectively [18]. Similarly, in a retrospective series of 282 patients from the Southeast United States, African-Americans accounted for 91% of the patients with collapsing FSGS and 15% of those with tip lesions [16]. In this series, patients with tip lesions were older with a mean (±SD) age of 54 (±13) years compared to a mean age of 38 (±12) years for the collapsing subtype [16].

The higher prevalence of collapsing FSGS in younger black adult patients and of tip lesions in older white adults was also, respectively, demonstrated in a cohort study from New York [15]. A series from Chicago similarly found tip lesion patients to be older but did not find significant differences in ethnic predilection based on histologic subtypes [14]. Importantly, this series did not distinguish the cellular from the collapsing variant, which may account for these slightly divergent results [14]. The impact of ethnicity on the relative prevalence of histologic variants was further demonstrated by two studies from India that reported FSGS NOS to be the most common variant. (44.6 to 72.5%) and the relatively low frequency of tip (12.3 to 13.5%) variant and collapsing variant (2 to 13.8%) [26, 27]. FSGS NOS was also the most common variant seen in one institution in China (55.9%) with tip and collapsing variants accounting for 4.8% and 6.9% of cases, respectively [28]. Paik et al. similarly found FSGS NOS to be the leading subtype identified in a Korean pediatric population [17].

### 3.2. Clinical Presentation

The clinical presentation can provide some clues to the histologic variant expected upon biopsy. Most studies have noted that tip lesion and collapsing FSGS are likely to present with nephrotic syndrome with the collapsing variant much more frequently associated with a reduced GFR [14–16, 18, 20, 25]. In contrast, there is a lower frequency of nephrotic syndrome in the perihilar subtype (25–55%) [16, 19]. It has similarly been observed that a smaller percentage of patients with NOS variant have nephrotic syndrome on presentation (57–67%) compared to the high rates reported for both tip and collapsing variants (80–90%), though the frequency of initial nephrotic syndrome may be greater for the NOS variant versus the perihilar variant [16]. Due to a relatively low prevalence, the cellular variant is often excluded from statistical analyses in retrospective or case series, which limits clinical characterization of this subgroup. However, a Columbia University case series noted that, like collapsing and tip lesion cases, patients with cellular FSGS were significantly more likely to present with nephrotic syndrome compared to the FSGS NOS subgroup (86% versus 53%, resp.) [15]. In addition, the Rush University group in Chicago found that a combined cellular/collapsing subgroup was more likely to present with nephrotic syndrome and massive proteinuria (>10 g/day) compared to a “classic” FSGS subgroup consisting of FSGS NOS and perihilar variants [14]. When compared to FSGS NOS, patients with collapsing, tip lesion, and cellular FSGS tend to present with shorter symptom duration [16, 26, 27]. The presence of hypertension at presentation seems more variable as some studies note a higher frequency in the collapsing subgroup [18, 19] while the University of North Carolina series found that hypertension was more commonly present in NOS and perihilar patients [16].

### 3.3. Biopsy Findings

Additional specific pathologic findings beyond histologic subtype classification may have important prognostic implications for FSGS patients. Collapsing FSGS cases have demonstrated significantly greater percentages of global sclerosis than tip lesion cases with higher numbers of segmental lesions and greater degree of overall glomerular involvement (global plus segmental lesions) than either cellular or tip lesion variants [15, 25]. The FSGS-CT noted a higher degree of globally sclerotic glomeruli in FSGS NOS compared to tip lesion subtype despite the younger age of the FSGS NOS population [20]. The Dutch series of adult FSGS patients found glomerular sclerosis and hyalinosis to be the most severe in the perihilar subgroup, intermediate in FSGS NOS subgroup, and the least severe in tip variant. Interestingly, electron microscopy exam of 51 patients showed that significantly more tip lesion cases displayed foot process effacement compared with FSGS NOS and perihilar FSGS [19]. Consistent with a reduced GFR on clinical presentation, collapsing FSGS cases have more tubulointerstitial injury than those with the tip variant [15, 18, 20, 29]. The prognostic importance of the extent of tubulointerstitial injury on biopsy was further demonstrated by the results of the FSGSCT. Here, while the presence of the collapsing variant, glomerulosclerosis progression, and tubulointerstitial injury were all significant predictors of ESRD, after adjustment for eGFR, age, and proteinuria, only percent tubular atrophy/interstitial fibrosis remained as a significant predictor (hazard ratio [95% confidence interval] = 1.21 [1.03, 1.42] per 10 percentage point increase, *P* = 0.02) [20].

### 3.4. Clinical Course

Classically, the tip lesion has been considered the most responsive to steroid therapy while the collapsing variant has been thought to be steroid-resistant and associated with a more aggressive clinical course. This has been largely validated by multiple series from diverse ethnic and demographic groups. In the University of North Carolina series, 50% of tip lesion patients achieved a complete remission after a median follow-up period of 1.8 years compared to 14% of collapsing and 13% of FSGS NOS cases [16]. In this study, renal outcomes were also better for tip lesion patients as they had a 3-year renal survival rate of 76% compared to only 33% for collapsing lesions and 65% for NOS cases [16]. The Columbia retrospective series also found the highest total remission rate (75.8%) and the lowest ESRD rate (5.7%) among the tip lesion subgroup after a mean follow-up period of 20 months, while the collapsing variant showed the lowest total remission rate (13.2%) and the highest ESRD rate (65.3%) [15]. Remission rates and renal outcomes were intermediate for cellular and NOS subgroups in this series [15]. While the tip lesion subgroup in the Netherlands series had the highest total remission rate (57%), this difference was not statistically significant compared with that of other subtypes, which perhaps may be reflective of the low frequency of collapsing cases as a basis for comparison [19]. However, five-year renal survival was still significantly better for tip variant (78%) than for FSGS NOS (63%) and perihilar (55%) subtypes in this study [19]. Interestingly, the spontaneous remission rate for the tip lesion subgroup was higher than the remission rate after immunosuppressive therapy (81% versus 43%), which supports the theory that tip variant may represent an FSGS/minimal change disease overlap [19]. Despite the absence of a tip variant group for comparison and initial low rates of steroid-induced remission overall in the New Orleans pediatric study, collapsing patients still had a significantly lower remission rate than FSGS NOS and cellular subtypes (45% versus 72.7% versus 75%, resp.) [18]. The collapsing subgroup also had the highest rate of progression to chronic kidney disease stage five (CKD 5), over a mean follow-up period of 3.9 ± 0.5 years [18]. Even among young steroid-resistant patients in the FSGS-CT, there were still histologically-based differences in renal outcomes, as collapsing FSGS patients had a significantly higher rate of progression to ESRD at 1 year (28%) and 3 years (47%) compared with FSGS NOS (1-year ESRD rate of 6%, 3-year ESRD rate of 20%) and tip lesion (1- and 3-year ESRD rates both of 7%) variants [20].

## 4. FSGS Recurrence after Transplantation

While renal transplantation is curative for many primary kidney disorders, FSGS has been found to recur at a disappointingly high rate of 20–40% after transplantation [30–33]. Compared to patients with recurrence of other glomerulonephritides, those with FSGS recurrence have a twofold higher risk of losing the graft over 10 years [34]. Risk factors for recurrent FSGS include younger age (especially in children <6 at FSGS onset), nonblack race, a rapid progression to ESRD in the native kidney (<3 years), heavy proteinuria in the period before transplantation, and the loss of previous allografts to recurrence [35, 36].

There have been interesting observations regarding the dynamic nature of histologic lesions seen in FSGS from the pre- to the posttransplant periods. In a multicenter study of 21 cases of recurrent FSGS, 81% occurred in the same pattern as the original disease, but, interestingly, three cases demonstrated a shift from FSGS NOS to collapsing, two collapsing to FSGS NOS, and one cellular to FSGS NOS [33]. As the authors highlight, there was interestingly no conversion between the cellular and collapsing subtypes after transplantation, which further supports the classification of these variants as distinct entities of FSGS. In addition, an intervening minimal change-like lesion was reported in 6 cases in the early (<1 month) posttransplant period [33]. Canaud et al. reported FSGS after transplant in 10 out of 10 children who had minimal change disease in the native kidney, and 30% of patients who had minimal change disease early after transplant (<3 months) developed FSGS on subsequent repeat biopsy [37]. In contrast to the findings of Ijpelaar, however, the native kidney histological diagnosis was predictive neither of the variant of FSGS identified in any subsequent allograft nor of the risk of FSGS recurrence overall [37]. It has been postulated that the observed divergent histological patterns between native kidney and allograft may be consequences of modification of FSGS-inducing circulating pathogenic factor by immunosuppressive medications, influence of the different genetic background of the donor kidney, and hemodynamic conditions that affect a solitary functioning donor kidney [37, 38].

Few studies in the available literature tested the prognostic power of Columbia classification in FSGS in the transplanted kidney. The ones published so far showed that posttransplant collapsing FSGS is associated with more severe vascular changes in renal allograft biopsy, higher degree of proteinuria, and renal insufficiency with higher rate of graft loss than other subtypes [39, 40]. However, more evidence is needed to establish the importance of histological subtype in the prognosis of recurrent or de novo FSGS. This information could be crucial for tailoring immunosuppression on a single patient basis.

## 5. Association of Histologic Classification with Immunohistochemical and Genetic Markers

Only few studies have correlated the histologic variants of FSGS with immunohistochemical expression of podocyte proteins. However, this research pathway is essential to improve our understanding of the pathophysiology of this condition, allowing identification of new diagnostic categories and distinct therapeutic approaches for each histologic variant.

During the embryonic stage, healthy podocytes divide normally, but, as they mature and differentiate, they leave the cell cycle and enter a quiescent adult state. Intriguingly, the podocytes from patients with collapsing variant of FSGS and HIV associated nephropathy (HIVAN) return to express the proliferation marker Ki-67 [41].

Recently, an immunohistochemical analysis (CD10, WT-1, Vimentin, Synaptopodin, *α*-actinin-4, GLEPP-1, cytokeratin (CK) 8-18, CK19, and Ki-67) of 131 renal biopsies with a diagnosis of primary FSGS classified according to the Columbia criteria was performed [42]. This study showed differences in podocyte differentiation and structural proteins in the variants of FSGS, which may have practical application. Collapsing variant of FSGS distinguished itself from others in terms of immunohistochemical expression of podocyte markers in injured glomeruli as it showed a higher occurrence of loss of expression of CD10, *α*-actinin-4, and WT1. Variants also presented differences in immunoexpression of CK8-18 and CK19 in podocytes of glomerular lesions [42]. This highlights the potential role of immunohistochemical markers in distinguishing FSGS variants. Additional and complementary methods, as well as larger series, are required to validate these findings.

It has been noted in the literature that genetic FSGS, while accounting for only a small percentage of adult-onset FSGS cases, tends to be resistant to treatment with steroids and calcineurin inhibitors, has a higher incidence of progression to ESRD, and is less likely to recur after transplantation than nongenetic FSGS cases [43, 44]. However, a possible association of genetic mutations with FSGS histologic subtypes has not yet been explored and thus would be an interesting inquiry for future investigation.

## 6. Conclusions

The Columbia histologic classification, since its original description 10 years ago, is a useful prognostic indicator in FSGS. Patients with the tip lesion tend to be older and Caucasian. Collapsing FSGS is seen more frequently in younger, black patients and has a high risk of progression to ESRD and the lowest remission rates.

Though some reports have suggested that the tip lesion can present with heavier proteinuria than FSGS NOS, it has a more indolent clinical course with higher rates of partial and complete remission and the lowest progression to ESRD. The difference in renal survival between the tip and collapsing lesions has been validated in the prospective FSGS-CT trial of young steroid nonresponders. Caution should be applied when using the classification to guide management decisions given the inherent limitations of biopsy sampling and variability in applying the criteria for each subtype. There is also now some evidence that the histologic criteria do not predict the risk of disease recurrence in renal allografts and, interestingly, different variants can be seen before and after transplant in the same patient. A mechanistic basis for morphologic differences noted pathologically also remains elusive. Future studies will be needed to identify reliable biomarkers to further define this disorder while making use of this valuable histologic classification.

## Figures and Tables

**Table 1:** Pathological features of different FSGS histologic variants [13].

Histological variant	Pathologic features
NOS	(i) Focal and segmental consolidation of the glomerular tuft by increased ECM^*^, leading to obliteration of glomerular capillary lumen (ii) May have segmental capillary wall collapse without overlying podocyte hyperplasia (iii) *Exclude perihilar, cellular, tip, and collapsing variants *

Perihilar	(i) At least 1 glomerulus with perihilar hyalinosis with or without sclerosis with >50% of sclerotic glomeruli possessing perihilar lesions (ii) Perihilar lesions located at glomerular vascular pole (iii) May be glomerular hypertrophy in adaptive FSGS (iv) *Exclude cellular, tip, and collapsing variants *

Cellular	(i) At least 1 glomerulus with endocapillary hypercellularity (including foam cells, macrophages, and endothelial cells) involving >25% of the glomerular tuft, leading to occlusion of the capillary lumen (ii) *Exclude tip and collapsing variants *

Tip	(i) At least 1 segmental lesion involving the “tip” domain (the outer portion of the glomerular tuft next to the origin of the proximal tubule) (ii) Either ECM adhesion or confluence of podocytes with parietal or tubular epithelial cells (iii) *Exclude collapsing variant and perihilar sclerosis/endocapillary hypercellularity *

Collapsing	(i) At least 1 glomerulus with collapse and overlying podocyte hypertrophy and hyperplasia (ii) Hyperplastic podocytes may fill urinary space, resembling crescents (iii) May have tubular injury and microcysts

^*^ECM = extracellular matrix.

**Table 2:** Summary of the main studies evaluating patient characteristics at presentation and renal outcomes of different FSGS variants.

Study (patients)	Age (years)	Ethnicity	Baseline nephrotic syndrome (%)	Baseline renal function	Remission (%)	Renal outcomes
Chun et al. 2004 [14] (*n* = 87)	NOS/perihilar: 40 ± 17 Cell + coll: 38 ± 16 Tip: 53 ± 17	% **Black race** NOS/perihilar: 47 Cell + coll: 72 Tip: 44	NOS/perihilar: 75 Cell + Coll: 84 Tip: 100	**Cr (mg/dl)**: NOS: 1.4 ± 0.5 Cell + coll: 1.9 ± 1.4 Tip: 1.6 ± 0.8	**CR/PR/CR + PR** NOS/perihilar: 16.7/13.9/30.6 Cell + coll: 15/30/45 Tip: 45.4/18.2/63.6	% **ESRD** NOS/perihilar: 25 Cell + coll: 43 Tip: 27

Stokes et al. 2006 [15] (*n* = 225)	All: 37.7 ± 1.3 NOS: 32.5 ± 2.0 Cell: 48.8 ± 4.9 Coll 31.2 ± 2.3 Tip: 47.5 ± 2.3	% **Black race** All: 31.5 NOS: 28.9 Cell: 31.8 Coll: 53.6 Tip: 13.8	All: 75.5 NOS: 52.9 Coll: 85.5 Cell: 86.4 Tip: 94.8	**Cr (mg/dl)**: All: 2.3 ± 0.2 NOS: 1.9 ± 0.2 Cell: 1.9 ± 0.3 Coll: 3.9 ± 0.6 Tip: 1.5 ± 0.1	**CR/PR/CR + PR** All: 27.3/11.2/38.5 NOS: 24.1/14.5/38.6 Cell: 38.9/5.5/44.4 Coll: 7.5/5.7/13.2 Tip: 60.6/15.2/75.8	% **ESRD** All: 30.7 NOS: 34.5 Cell: 27.8 Coll: 65.3 Tip: 5.7

Thomas et al. 2006 [16] (*n* = 197)	All: 49 ± 15 NOS: 50 ± 15 Cell: 45 ± 13 Coll: 38 ± 12 Tip: 54 ± 13 Perihilar: 50 ± 16	% **Black race** All: 40 NOS: 43 Cell: 33 Coll: 91 Tip: 15 Perihilar: 29	All: 70 NOS: 67 Cell: 75 Coll: 83 Tip: 88 Perihilar: 55	**Cr (mg/dl)**: All: 2.1 ± 2.0 NOS: 2.1 ± 1.8 Cell: 2.5 ± 1.7 Coll: 3.1 ± 3.8 Tip: 1.5 ± 0.9 Perihilar: 2.0 ± 1.4	**CR/(CR or PR)** All: 19/24 NOS: 13/16 Cell: 33/33 Coll: 14/18 Tip: 50/53 Perihilar: 10/19	% **Renal survival** ^+^ ** 3-year** All: 67 NOS: 65 Cell: NA Coll: 33 Tip: 76 Perihilar: 75

Paik et al. 2007 [17] (*n* = 92)	All: 6.7 ± 3.5	N/A	All: 92.4	**Cr (mg/dl)**: All: 0.77 ± 0.50	**CR/PR** All: 39.1/15.2/54.3	% ESRD All: 21.7% % **Renal survival** ^‡^ **5-year**: 84 **10-year**: 64 **15-year**: 53

Silverstein and Craver 2007 [18] (*n* = 41)	All: 10.9 ± 0.9	% **Black race** All: 80.5%	All: 63.4 NOS: 55.6 Cell: 81.8 Coll: 54.5	**eGFR (ml/min/1.73 m** ^ 2^ **)** All: 105.4 ± 4.6 NOS: 116.5 ± 4.9 Cell: 107.6 ± 7.2 Coll: 88.2 ± 10.7	**CR + PR** All: 71 NOS: 72.2 Cell: 75 Coll: 45.4	% **CKD stage 5** NOS: 0 Cell: 0 Coll: 18.2

Deegens et al. 2008 [19] (*n* = 93)	All: 49 ± 16 NOS: 51 ± 17 Collapsing: 63 ± 18 Tip: 44 ± 16 Perihilar: 50 ± 12	% **Dutch descent**: All: 96 NOS: 93 Collapsing: 100 Tip: 97 Perihilar: 96	% **NS: ** All: 63 NOS: 57 Collapsing: 80 Tip: 97 Perihilar: 25	**Cr (mg/dl)**: All: 1.7 ± 1.1 NOS: 1.99 ± 1.3 Collapsing: 2.2 ± 1.6 Tip: 1.3 ± 0.9 Perihilar: 1.6 ± 0.99	**CR + PR** All: 44 NOS: 38 Collapsing: 50 Tip: 57 Perihilar: 25	% **Renal survival** ^~^ **5-year** All: 66 NOS: 63 Collapsing: 30 Tip: 78 Perihilar: 55

D'Agati et al. 2013 [20] (*n* = 138)	NOS: 13 Coll: 16.5 Tip: 15	% **Black race**: NOS: 39 Coll: 63 Tip: 14	% **severe/intermed. nephrosis** ^*^: NOS: 69 Tip: 93 Coll: 94	**Cr (mg/dl)**: NOS: 0.86 ± 0.61 Tip: 0.84 ± 0.43 Coll: 1.33 ± 0.50	**N/A**	% **ESRD at 3 yrs** NOS: 20 Coll: 47 Tip: 7

^*^Intermediate nephrosis = U p/c > 2 and ≤6 g/g or serum albumin ≥1.5 and <2.5 g/dl; severe nephrosis = Up/c > 6 g/g or serum albumin <1.5 g/dl.

^+^Renal failure = sustained doubling of the serum creatinine or initiation of chronic dialysis or kidney transplantation.

^‡^Renal survival = measured from date of presentation to diagnosis of CRF.

^~^Renal failure = sustained increase of the serum Cr concentration of >50% from baseline or development of ESRD.
